# Epidemiological profile trends and cost of pediatric sickle cell disease in Brazil from 2008 to 2022

**DOI:** 10.1016/j.jped.2024.07.010

**Published:** 2024-09-07

**Authors:** Luiza Telles, Paulo Henrique Moreira Melo, Luana Baptistele Dornelas, Gabriele Eckerdt Lech, Natália Zaneti Sampaio, Ayla Gerk, Madeleine Carroll, Cristina Pires Camargo

**Affiliations:** a Instituto de Educação Médica (IDOMED/Estácio, Campus Vista Carioca), Rio de Janeiro, RJ, Brazil; b Universidade Federal de Minas Gerais, Faculdade de Medicina, Belo Horizonte, MG, Brazil; c Faculdade de Ciências Médicas da Santa Casa de São Paulo, São Paulo, SP, Brazil; d Pontifícia Universidade Católica do Rio Grande do Sul, Porto Alegre, RS, Brazil; e Universidade de Araraquara, Araraquara, SP, Brazil; f Harvard Medical School, Program in Global Surgery and Social Change, Boston, United States; g McGill University, Department of Surgical and Interventional Sciences, Quebec, Canada; h Montreal Children's Hospital, Harvey E. Beardmore Division of Pediatric Surgery, Quebec, Canada; i Faculdade de Medicina, Universidade de São Paulo, Microcirurgia Laboratorial e Cirurgia Plástica, São Paulo, SP, Brazil

**Keywords:** Public health, Pediatric, Sickle cell anemia, Global health, Cost of illness

## Abstract

**Objective:**

This study aimed to investigate the epidemiological trends of Pediatric Sickle Cell Disease (SCD) in Brazil over the period 2008–2022, with a focus on understanding the incidence, mortality rates, and associated healthcare costs. The study explored potential associations between patient characteristics and the occurrence of crises in pediatric SCD cases.

**Methods:**

A cross-sectional study was conducted, analyzing national annual rates of pediatric SCD hospitalizations using data from the FioCruz platform. Descriptive and inferential analyses, including time series and ARIMA regression, were employed. Economic dimensions were assessed using cost categorization. The study followed STROBE reporting guidelines.

**Results:**

Data on 81,942 pediatric SCD hospitalizations were collected, with a predominance of crisis-related cases (74.08 %). Males and children under five years old were most affected. Regional disparities were observed, with the Southwest region recording the highest hospitalization rates. ICU costs were higher for crisis-related hospitalizations. Mortality rates were significantly higher for crisis-related cases (*p* < 0.001), with ARIMA regression indicating a significant association between hospitalizations for crisis-related cases and mortality.

**Conclusion:**

This study highlights the significant burden of pediatric SCD in Brazil, particularly crisis-related cases, suggesting a need for focused interventions. By prioritizing early detection, equitable access to healthcare, and evidence-based interventions, Brazil can mitigate the burden of SCD and improve patient outcomes. These findings contribute to informing public health policies and interventions aimed at addressing the challenges of pediatric SCD management in Brazil.

## Introduction

Sickle cell disease (SCD) is one of the most frequent autosomal-recessive genetic diseases from abnormal hemoglobin.^1^ This condition can lead to blood vessel occlusion, resulting in acute vaso-occlusive crises and chronic ischemic disorders. Acute vaso-occlusive crises are characterized by sudden, severe pain, while chronic ischemic disorders can manifest as hemolytic anemia or extensive organ damage.^2^

In Brazil, an estimated 3500 children are born annually with sickle cell disease (SCD). As a measure to monitor the disease, SCD has been included in the Brazilian National Neonatal Screening Program (NNSP), coordinated by the Ministry of Health since 2001. This program covers all 26 states and the Federal District. It aims to address SCD and other hemoglobinopathies^1^ by regulating access to care and overseeing authorization, registration, and reimbursement procedures.

The clinical manifestations usually begin after three months of age and persist throughout the individual's lifespan.^3^ Also, SCD can cause complications, such as splenic sequestration crises, especially in patients under five years old, and death.^4^ The literature shows a death percentage of pediatric patients with SCD, in Brazil, of 7.4 %.^5^

Moreover, beyond the health concerns related to mortality rates due to SCD, its complications pose an economic challenge to the Brazilian Health System. For instance, vaso-occlusive crises, the complication with the highest financial burden, cost around 11,410,960 USD annually in pediatric health system financing, according to research conducted by Silva-Pinto et al.^6^ Despite ongoing efforts to enhance early detection and management of SCD, challenges persist in disease management within Brazil. This study evaluates the national trends in Pediatric SCD, assessing Brazil's incidence, mortality, and public health system costs from 2008 to 2022. Additionally, it investigates the differences in health patterns between patients experiencing crises and those who do not.

## Methods

This cross-sectional study evaluated the national annual rates of SCD for pediatric patients in Brazil. From January 2008 to December 2022, data was retrieved from the FioCruz platform, “Plataforma de Ciência de Dados Aplicada à Saúde” (PCDaS).^1^^,^^7^ PCDaS is a national, open-access Brazilian discharge database that provides health-related information for patients admitted to the Universal Health System (SUS), which includes public and private health data. The Data Science Platform for Health (Plataforma de Ciência de Dados aplicada à Saúde, PCDaS) is an initiative by the Health Information Laboratory (Laboratório de Informação em Saúde, Lis) of the Institute for Scientific and Technological Communication and Information in Health (Instituto de Comunicação e Informação Científica e Tecnológica em Saúde, Icict) at the Oswaldo Cruz Foundation (Fundação Oswaldo Cruz, Fiocruz). The primary goal of PCDaS is to provide technological services and scientific computing for storing, managing, and analyzing large data volumes to researchers, faculty, and students from educational and research institutions. It provides anonymized patient data to all basic and academic users.

The authors accessed the "Sistema de Informações Hospitalares do SUS" (SUS Hospitalar Healthcare Information System) in chapter XIX of this platform. The authors collected data using the International Classification of Diseases (ICD-10) chapter III codes for Sickle Cell Disorders (D57), specifically those for Sickle Cell crises (D57.0) and Sickle Cell without crises (D57.1). The pediatric population was defined as under 18 years old and analyzed separately from the adult population. The annual estimates were incidence by sex, age, Brazilian regions, procedures performed in those patients, and ICU admissions. The mortality rate was also assessed. The present study followed the Strengthening the Reporting of Observational Studies in Epidemiology (STROBE) reporting guidelines.^8^ The cost assessed serves as the national reference for hospitals to receive payment for treatment performed and professional services related to patient care. This cost includes all professional and hospital services involved in patient care, such as surgeons, anesthesiologists, nursery, surgical materials and equipment, daily rates, medication, room fees, and meals provided in Unified Health System (SUS)-affiliated hospitals.

### Statistical analysis

#### Data representation

The descriptive analysis represented parametric data using mean and standard deviation, non-parametric data by median and interquartile range, and proportions by percentage.

#### Inferential analysis

For inferential analysis, the study employed a time-series analysis utilizing time-series graphics, and ARIMA was employed for regression analysis.

#### Cost analysis

To analyze the economic dimensions, hospital costs were categorized in US dollars as follows: 0–100, 100–500, 500–1000, 1000–5000, and > 5000.

#### Statistical parameters

The study adhered to a significance level (alpha) of 5 % with a study power of 80 %. Statistical analyses were performed using STATA v18 (StataCorp. 2023. Stata Statistical Software: Release 18. College Station, TX: StataCorp LLC).

#### Ethical considerations

The study used publicly accessible secondary data online from the FioCruz database, PcDaS, and adhered to the International Guidelines for the Development of Research Involving Human Subjects. Consequently, it is exempt from formal ethical procedures.

## Results

The authors collected data on 81,942 hospitalizations due to SCD from 2008 to 2022 in the pediatric population. Among these cases, 60,707 (74.09 %) were associated with sickle cell anemia with crises, while 21,235 (25.91 %) were associated with sickle cell anemia without crises. Both hospitalizations for sickle cell anemia with crises and those for sickle cell anemia without crises exhibited a slight predominance of the male population. Specifically, 52.67 % of hospitalizations for sickle cell anemia with crises were male patients, while 50.18 % of hospitalizations for sickle cell anemia without crises were male patients. When analyzing the data according to the age of the patients, SCD with and without crises was most predominant among children from 0 to 5 years old, presenting 31,699 and 8778 cases, respectively. Between the ages of 5 and 10 years old, 5791 children were admitted without crises, while 25,337 were admitted with a crisis. This trend declined in the 10–15 age group, with only 4448 admissions without crises and 20,026 with crises.

Concerning types of treatments performed, 56,361 patients with crises and 19,041 without crises were treated for hemolytic anemia. It was followed by diagnosis or urgent care in pediatrics, aplastic anemia, or other anemia treatment. When analyzing the surgical beds, 89 splenectomies were performed, followed by 66 urgent surgical care evaluations, and 29 treatments with multiple surgeries. The analysis of incidence by regions showed that the Southeast reached the highest number of SCD for both with and without crises, presenting 30,272 and 13,687 cases, respectively. The lowest number in both cases was addressed to the South, with 2798 cases of SCD with crises and 686 without crises.

**Figure 1:**
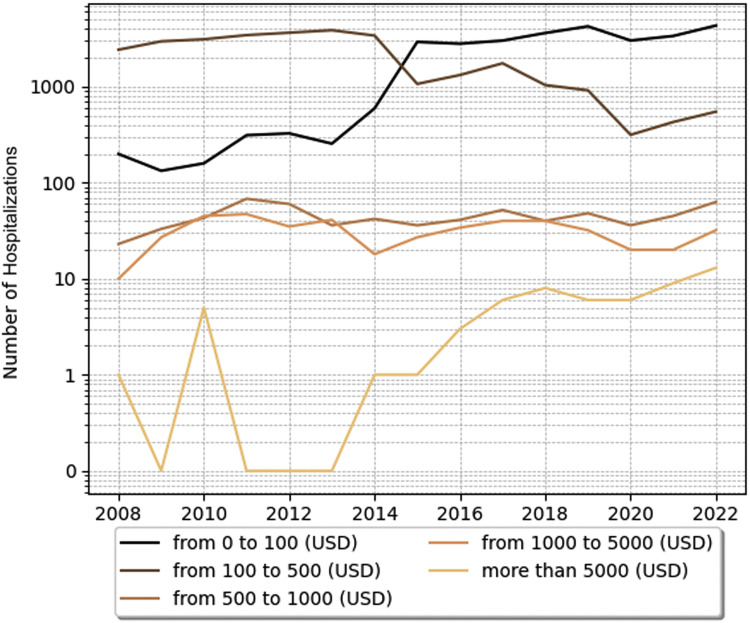
Annual trends in hospitalizations of SCD pediatric patients with crisis costs across different price categories. SCD, sickle cell disease; USD, United States dollar.

**Figure 2:**
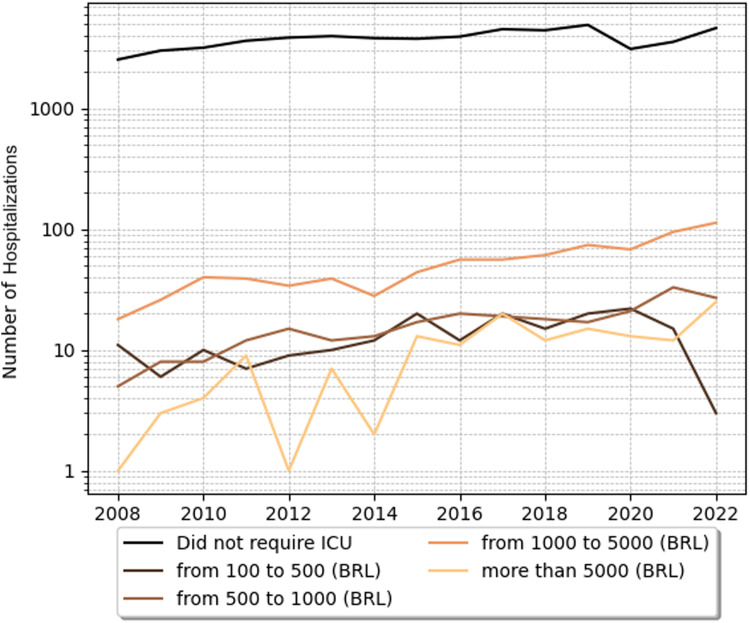
Annual trends in ICU bed costs of sickle cell disease with crises across different price categories. BRL, Brazilian real; ICU, Intensive care unit; SCD, Sickle cell disease.

**Figure 3:**
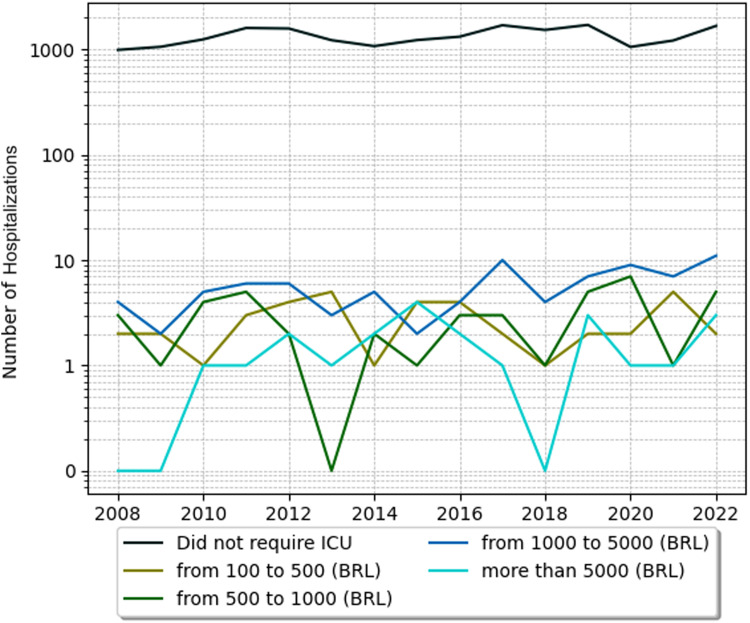
Annual trends in ICU bed costs of sickle cell disease without crises across different price categories. BRL, Brazilian real; ICU, Intensive care unit; SCD, Sickle cell disease.

The costs of intensive care unit (ICU) stays were also analyzed. In SCD pediatric patients with crises ICU hospitalizations, costs were higher, with the mean cost of hospitalization for each year ranging from 377.43 USD (1885.15 BRL, considering the latest dollar price of 5 BRL- Brazilian currency) to 577.56 USD (2884.97 BRL). In a time analysis, those patients had higher costs in ICU in 2021 and 2022, as shown in Figure 1, Figure 2, Figure 3. Costs from SCD patients without crises in the ICU were lower - from around 294.00 USD (1468.56 BRL) to 536.00 USD (2677.37 BRL).

As to mortality, this analysis showed an average of 76 deaths per year among patients hospitalized with crises, with a 95 % confidence interval between 76 ± 21.5 (64.08 and 87.92; 95 % CI). For those without crises, the mean annual death count was 21.2, with a confidence interval ranging from 21.2 ± 6.1 (2031.4–2509.9; 95 % CI).

**Figure 4:**
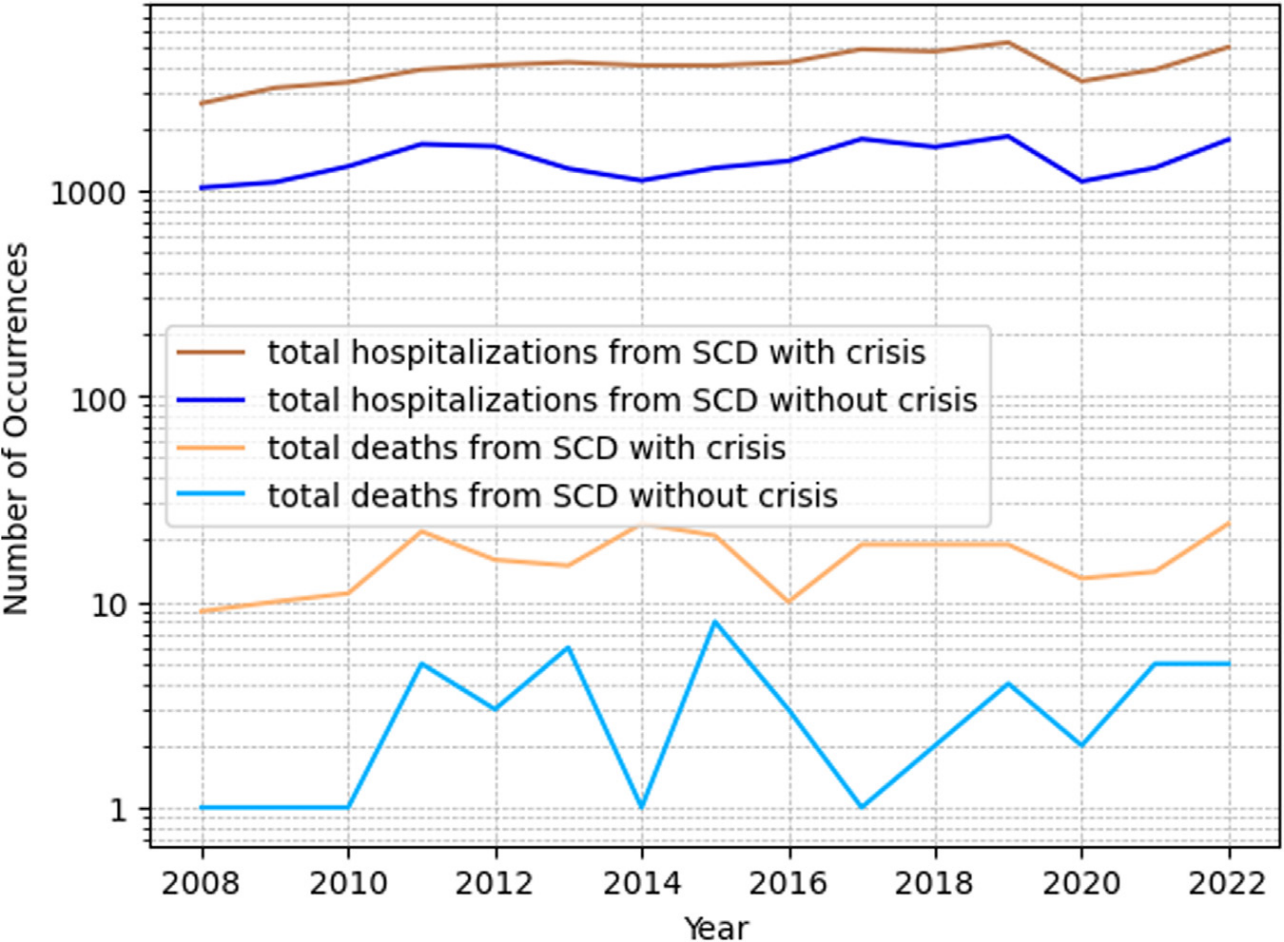
Hospitalizations and deaths from sickle cell disorders. SCD, sickle cell disease.

The statistical significance of the differences in hospitalization and mortality rates for cases with crises compared to those without crises was confirmed with a p-value<0.001. This level of significance, well below the 0.01 % threshold, robustly suggests that the observed differences are not due to random variation but represent a genuine disparity in the data. The results of hospitalizations and deaths from SCD are registered in Figure 4.

A time series regression method known as ARIMA (AutoRegressive Integrated Moving Average) revealed a significant relationship between patients hospitalized for sickle cell anemia with crises and mortality, with a p-value of 0.004 and a 95 % confidence interval (11.6 - 59.36), and a coefficient of 35.5. In contrast, no significant relationship was found between hospitalizations for sickle cell anemia without crises and mortality. A p-value of 0.015, a 95 % confidence interval (CI) of 6.5 to 60.4, and a coefficient of 33.4 showed that there was a strong association between hospitalizations of patients with anemia and death.

## Discussion

In the present study of 96,079 pediatric hospitalizations due to sickle cell disease, 63.1 % were in crises and 36.9 % were without crises, indicating a higher prevalence of crises-related hospitalizations. Although limited data is available, current literature suggests that many of these crisis-related hospitalizations may be related to inadequate pain management.^9^, ^10^, ^11^, ^12^, ^13^, ^14^

Regarding mortality, the present findings show that SCD patients in crises had an average of 18.78 deaths per 1000 patients, per year, whereas those without crises had an average of 14.98 deaths per 1000 patients per year. Demographically, a higher prevalence of SCD was observed in males and children up to 5 years old. Similar trends are observed in existing literature. An ecological study conducted from 2000 to 2019 revealed a higher frequency of mortality among males aged zero to nine (55.2 %).^15^

Additionally, the present study identified significant regional disparities in SCD prevalence and outcomes across Brazil. Although the Southwest region recorded the highest absolute number of SCD cases in the present research, literature traditionally shows the Northeast region having the highest number of deaths.^15^ This disparity may be attributed to several factors in the Southeast region of Brazil, which boasts a higher number of hospital beds, attracts patients from other regions seeking better treatment, and has superior healthcare indicators compared to the Northeast, including lower child mortality rates and a more robust healthcare infrastructure.^16^^,^^17^ Also, environmental determinants for SCD severity may be associated with this region's prevalence difference.^18^

Concerning race, the majority of victims were black (78.73 %) in previous literature;^15^ however, this information was not available for analysis. Similarly, the literature indicates a higher prevalence of SCD in both adult and pediatric black patients.^19^ These findings may be attributed to structural racism and economic inequality, which impact access to medical assistance and disease management.^13^

Understanding the epidemiological pattern is also crucial for public health financing. The present results evidence the economic burden of the SCD crises. ICU stay costs for patients in crises varied from 377.43 USD to 577.56 USD, whereas for patients without crises, they varied from 294.00 USD to 536.00 USD. Comparatively, Silva-Pinto et al. found that the average standard care costs for acute complications in Brazil were 1835 USD and 987 USD for adults and children, respectively. For chronic complications, the costs were 769 USD and 116 USD, annually totaling over 413 million USD for SCD treatment.^6^ The higher costs associated with crises can be attributed to the complexity of symptom management and the conditions in a hospital setting. In this scenario, implementing strategies to prevent symptom exacerbations can reduce long-term costs associated with vaso-occlusion episodes, hemolytic anemia, and vasculopathy.^6^^,^^20^^,^^21^

Perhaps, comprehensive care strategies, including the prescription of prophylactic penicillin for children, iron chelators, and hydroxyurea, are instrumental in preventing hospitalizations due to sickle cell crises. Strategies targeting symptom prevention and adherence to pain management guidelines are crucial for reducing healthcare costs and long-term complications. These measures, along with adherence to WHO guidelines for pain management, can significantly reduce the frequency of crisis episodes, thus decreasing the need for hospitalization in pediatric SCD patients. These strategies not only address acute management but also contribute to long-term health benefits, reducing both the physical and economic burdens of SCD.^22^

This study has its limitations. Firstly, the data used for the analysis is from a public database and relies on the classification of diseases, procedures, and treatments. Therefore, the total number of patients of interest may be underestimated due to potential errors in hospital classification, which can lead to inaccuracies in published data. Another constraint is that it is not possible to distinguish multiple admissions of the same patient. Furthermore, some treatments obtained from the database can be generalized or broadly classified. Secondly, the authors utilized only hospitalization data, which might not reflect the true incidence of the disease due to the potential for ambulatory treatment and migration of patients to other Brazilian regions to receive better healthcare. Thirdly, the exclusion of data about other sickle cell disorders may impact the generalizability of the results. Despite these factors introducing bias in the present results, statistical analysis with confidence intervals of 95 % and evidence of statistical significance help mitigate these limitations.

## Conclusion

This study has highlighted a significant number of hospital admissions related to sickle cell disease (SCD), with cases involving crises predominating, especially among males and children under five years old. Regional disparities are apparent, with the Southeast region showing the highest absolute numbers of hospitalizations. Focused strategies on symptom prevention and adherence to pain management guidelines are essential for reducing both immediate healthcare costs and long-term complications. By emphasizing early detection, ensuring equitable access to healthcare, and implementing evidence-based interventions, the authors can effectively reduce the burden of SCD and enhance patient outcomes across Brazil.

## Authors’ contributions

LT, PHMM, GEL, and CC conceptualized and defined the manuscript's methodology, and described the funding acquisition and resources. LT did the project administration. LT, PHMM, GEL, LBD, NZS, AG, MC, and CC participated in software used management, and data curation, validated the research outputs, and did the investigation. LT, PHMM, GEL, LBD, NZS, AG, MC, and CC performed the formal analysis and wrote the original draft. MC and CC reviewed and critically edited and visualized the final manuscript. CC supervised and validated the manuscript.

## Abbreviations

SCD, Sickle Cell Disease; Brazilian National Neonatal Screening Program (NNSP).

## Conflicts of interest

The authors declare no conflicts of interest.
